# Improving Human Papillomavirus (HPV) Vaccine Uptake in Canada: A Call for Equity and Inclusivity

**DOI:** 10.1177/2752535X251368427

**Published:** 2025-08-14

**Authors:** Noah Doucette, Audrey Steenbeek

**Affiliations:** 1 3688 School of Nursing, Dalhousie University, Halifax, Nova Scotia, Canada

**Keywords:** human papillomavirus, vaccination, normalization, health equity, inclusivity, disparities

## Abstract

Human Papillomavirus (HPV) is the most common sexually transmitted infection worldwide. Specifically, HPV is responsible for a large proportion of anal, cervical, vaginal, vulvar, penile and oropharyngeal cancers, highlighting the importance of optimizing the prevention of this public health issue. To date, vaccination is the most effective method for preventing HPV-related infections and associated diseases; however, vaccine uptake remains well below national targets. In Canada, gender-neutral HPV vaccination is recommended for all individuals between nine and 26 years, but can also be administered to adults until the age of 45. Despite widespread adoption of publicly-funded school-based vaccination programs, some populations report disproportionately lower rates of HPV vaccine uptake, including young adults, transgender peoples and men who have sex with men (MSM), rendering them vulnerable to morbidity and mortality. Addressing HPV-related disparities requires a coordinated, multi-level call to action involving collaboration between academic and community partners to normalize inclusive, gender-neutral vaccination. This paper explores opportunities for optimizing HPV vaccine uptake in Canada by emphasizing the importance of healthcare provider recommendation, improved access to community-based vaccination services, and representation of diverse populations (e.g., young adults, transgender peoples, MSM) in the development and delivery of vaccine communication/messaging. The time is now to normalize inclusive HPV vaccination in order to mitigate the persistence of vaccine-related disparities and strive toward global initiatives of health equity.

## Introduction

Human Papillomavirus (HPV) is the most common sexually transmitted infection worldwide, with an estimated 80% of sexually active individuals acquiring the virus at some point in their lifetime.^1,2^ While the majority of HPV infections are transient, high-risk genotypes, particularly HPV 16 and 18, were responsible for more than 690,000 new cancer diagnoses globally in 2018.^3^ Existing evidence suggests that HPV is known to cause more than 90% of anal and cervical cancers, 70% of oropharyngeal and vaginal cancers, and 60% of vulvar and penile cancers,^4^ highlighting the importance of optimizing the prevention of this pervasive public health issue.

Vaccination remains one of public health’s greatest achievements, effectively reducing morbidity and mortality from infectious diseases worldwide.^5^ Coupled with surveillance and screening, vaccination stands as the cornerstone to HPV prevention.^6^ In May 2018, the World Health Organization’s Director-General issued a global call to eliminate cervical cancer, emphasizing the need to vaccinate 90% of 15-year-old girls, screen 70% of women by ages 35 and 45 (respectively), and treat 90% of women with pre-cancerous and cancerous HPV by 2030.^7^ Despite this recommendation, rates of cervical cancer in Canada are increasing by approximately 3.7% per year, marking its first significant increase since 1984.^8^ Furthermore, HPV-related oropharyngeal cancers are also increasing, with rates being 4.5 times more prevalent in males than females, emphasizing the importance of gender-neutral vaccination.^9,10^

Historically, societal narratives have framed HPV as predominantly a women’s health issue, resulting in a gendered approach to vaccination.^11^ While widespread vaccine uptake in females has been proposed as a way of achieving herd immunity, this approach is inherently limited; it renders males vulnerable to HPV infections and fails to protect populations with low vaccine uptake, including young adults who were not eligible for school-based vaccination, transgender individuals and men who have sex with men (MSM).^9^ According to a systematic review and meta-analysis exploring HPV vaccine uptake in Canada, only 56% of individuals reported being vaccinated against HPV in 2017,^1^ which underscores the importance of mitigating the multi-level barriers that exacerbates the persistence of this public health issue.

This paper will discuss the need to normalize inclusive HPV vaccination in Canada by: (1) briefly outlining the evolution of HPV vaccination, (2) identifying disparities in HPV vaccine uptake, and (3) discussing recommendations to enhance HPV vaccine delivery by optimizing healthcare provider (HCP) recommendation, improving community-based vaccination services, and refining HPV vaccine communication/messaging.

## The Evolution of HPV Vaccination in Canada

Vaccination is the most effective intervention known to prevent HPV-related infections and associated diseases.^6^ Although HPV vaccination does not offer absolute protection from the virus, it can significantly reduce the prevalence of genotypes that are responsible for the majority of anogenital and oropharyngeal cancers.^6^ A Danish cohort study demonstrated an 86% reduction in cervical cancer incidence amongst females vaccinated prior to the age of 17,^12^ while a randomized control trial reported between 97 and 99% seroconversion for vaccine-targeted HPV genotypes in males, further supporting the vaccine’s efficacy in preventing incident and persistent infections amongst both, males and females.^13^

Currently, two vaccines are licensed for use in Canada: the bivalent (2vHPV) vaccine, which is available for females aged 9 to 45 years, and the nonavalent vaccine (9vHPV), which is licensed for both females and males within the same age demographic.^6^ In 2007/08, Canada was one of the first countries to implement publicly-funded school-based HPV vaccination programs, initially targeting girls due to early evidence suggesting cervical cancer prevention.^14^ Over time, gender-neutral vaccination programs were introduced between 2013/14 and 2018/19 (provincially and territorially dependent) to mitigate the burden of HPV-related infections and associated diseases more equitably.^14^ The National Advisory Committee on Immunization (NACI) now recommends HPV vaccination for all individuals between nine and 26 years, with safe administration for adults until the age of 45.^6^ Beyond school-based gender-neutral vaccination programs, most Canadian jurisdictions offer publicly-funded catch-up vaccination for high-risk populations, including transgender peoples, MSM and immunocompromised individuals (e.g., those living with the Human Immunodeficiency Virus).^14,15^

## Exploring Disparities to HPV Vaccine Uptake in Canada

Despite efforts to increase HPV vaccination coverage in Canada, rates remain below the NACI’s 90% target uptake goal.^6^ While a comprehensive exploration of population-specific disparities is beyond the scope of this paper, we will explore HPV vaccine uptake amongst key Canadian sub-populations, including young adults, transgender peoples and MSM.

### Catch-Up Vaccination Amongst Young Adults: Expanding the Scope Beyond Adolescents

Initiating vaccination prior to the onset of sexual activity remains a key determinant of HPV vaccine effectiveness.^16^ Pre-licensure clinical trials have consistently demonstrated that vaccine uptake preceding HPV exposure provides the greatest level of protection, emphasizing the importance of vaccinating youth.^16^ In Canada, school-based vaccination programs have significantly contributed to widespread vaccine uptake,^16^ with 76% of 14-year-olds in participating provinces and territories receiving at least one dose of the HPV vaccine in 2023.^6^ Similarly, a Canadian systematic review and meta-analysis reported approximately 70% uptake amongst youth in school-based programs, compared to only 19% in community settings (e.g., primary care vaccination).^1^ Conversely, vaccine uptake amongst adults (18 years of age and older) lags significantly behind, with only 15% reporting being vaccinated against HPV in 2023.^17^ According to the adult National Immunization Coverage Survey conducted in 2023, higher rates of vaccine uptake were observed in adults aged 18–26 years (62.9%), compared to those 27 years and older (12.5%).^17^

Despite publicly-funded HPV vaccination in Canada, existing public health strategies (e.g., knowledge campaigns) center predominantly around school-based programs, with little emphasis on catch-up vaccination amongst young adults until the age of 26 years.^18^ Although most effective prior to the onset of sexual activity,^16^ HPV vaccination remains essential for preventing infections amongst those who were not eligible, or missed school-based programs (e.g., parental hesitancy, delayed implementation),^18^ as the vaccine can still protect against HPV genotypes they may have not yet been exposed to.^6^ Additionally, due to frequent risky sexual behavior (e.g., multiple partners, unprotected sex) amongst young adults,^18,19^ increasing catch-up vaccination serves as a critical pathway to improve vaccine uptake, and mitigate the burden of HPV-related infections and associated diseases. According to a recent systematic review exploring catch-up HPV vaccination amongst young adult males- a population who remains disproportionately under-vaccinated compared to females-, health literacy, cost of the vaccine, absence of HCPs recommendation, as well as vaccine safety, efficacy and distrust were identified as barriers to uptake.^19^ Young adults are uniquely positioned to make informed health-related decisions,^18^ highlighting the importance of optimizing the delivery of inclusive HPV vaccination amongst this age demographic.

### Transgender Peoples: Addressing Gaps in HPV Vaccination

Transgender peoples face significant barriers to accessing affirming and competent health services, including preventative care like HPV vaccination.^20,21^ Despite many Canadian jurisdictions extending publicly-funded HPV vaccination to include transgender peoples, data on vaccine uptake within this population remains scarce.^20,21^ Transgender men (TM)- individuals assigned female at birth, who identify as male- are particularly vulnerable to HPV, experiencing comparable rates of infection and cervical cancer as cisgender women.^20,21^ In Canada however, only 56% of TM reported routine cervical cancer screening in 2016,^21^ with only 66% receiving at least one dose of the HPV vaccine in an American study in 2019.^22^ Furthermore, transgender women (TW)- individuals assigned male at birth, who identify as female- represent another population facing disproportionately lower rates of HPV vaccine uptake, with one American study reporting only 26% uptake amongst a national sample of 18- to 30-year-olds in 2019.^22^ Gaps in HPV prevention amongst transgender peoples further highlights the importance of revising existing public health strategies to extend beyond binary, gendered approaches (men and women) to HPV vaccination.^20^

Despite limited research efforts and representation in Canadian vaccination databases, transgender peoples face disproportionately higher rates of stigma and discrimination, limited access to health services, and suboptimal gender-affirming care, which can negatively influence vaccine uptake.^22^ Similarly, in a systematic review exploring COVID-19 vaccine hesitancy amongst members of the LGBTQ + population, previous negative healthcare experiences resulted in 25% of transgender peoples delaying vaccination, which can significantly increase their susceptibility to morbidity and mortality amidst a global pandemic.^23^ In order to mitigate persistent vaccine-related inequities amongst transgender peoples, it is imperative that public health policies, practices and research prioritizes building trusting relationships and safer healthcare environments that take into consideration the unique needs of this underserved population.^22,23^ If not adequately addressed, we risk exacerbating health inequities and the longstanding systemic injustices experienced by transgender communities. Normalizing inclusive HPV vaccination is a critical step towards increasing vaccine equity, and reducing the burden of HPV-related infections and associated diseases at the public health level.

### MSM: A High-Risk Population

While widespread HPV vaccine uptake amongst women can confer herd immunity to heterosexual men, this protection cannot be extended to MSM, who remain disproportionately impacted by adverse HPV-related health outcomes (e.g., anal cancer).^11,24^ A 2021 systematic review and meta-analysis of 107 studies (n = 36,773 MSM) revealed an overall pooled prevalence of anal HPV at 78%, penile HPV at 36%, oral HPV at 17%, and urethral HPV at 15%,^25^ highlighting the significant burden of infection amongst this targeted, high-risk population. To date, most Canadian provinces and territories have extended publicly-funded HPV vaccination programs to include MSM; however, eligibility, access, and uptake vary across jurisdictions.^14,15^ A cross-sectional study of Canada’s three largest cities (Toronto, Montreal and Vancouver) reported rates of HPV vaccine uptake between 14 to 35% for young MSM (26 years of age and younger) and 2 to 26% for older MSM (27 years of age and older),^26^ which remains significantly below national targets.^1^ This stark contrast in rates of HPV vaccine uptake amongst MSM highlights the importance of developing inclusive public health interventions aimed at increasing vaccination coverage amongst this demographic.

Despite targeted HPV vaccine recommendations for MSM, multi-level marginalization, stigmatization, homophobia and social influences (e.g., framing HPV as predominantly a women’s health issue) continue to negatively influence vaccine uptake.^27^ In a systematic review and meta-analysis exploring vaccine acceptability amongst MSM, several barriers were found to impede vaccination, including socioeconomic disadvantage (e.g., lower levels of education, living in a rural community, less annual income), restricted access and utilization of healthcare services (e.g., sexual health, primary care), suboptimal vaccine-related knowledge and attitudes, and fear of disclosing sexual orientation/practices to HCPs.^27^ In order to increase HPV vaccine uptake amongst MSM, it is imperative that public health initiatives (e.g., policies, programs, practices) consider the barriers to vaccine accessibility, the late adoption of males to HPV vaccination programs, as well as the multi-level barriers that reinforce longstanding inequities amongst this underserved population.^27^

## Recommendations for Optimizing HPV Vaccine Uptake

### Increasing Healthcare Provider (HCP) Recommendation

Healthcare providers are considered the most trusted source of information about vaccination, with their recommendation serving as a key predictor of vaccine uptake.^6,28^ However, many HCPs fail to provide effective recommendations for routine vaccination with their patients.^28^ Specifically, HCPs are challenged by the high cost of HPV vaccination for individuals not eligible for publicly-funded programs, and limited evidence-based guidelines for vaccinating high-risk and targeted populations (e.g., MSM, transgender peoples).^29^ For example, in a study exploring communication practices as a barrier to HPV vaccine uptake amongst MSM, approximately 70% of primary care practitioners were aware of existing vaccine recommendations, but only 13% routinely discussed sexual orientation and HPV vaccination.^30^ Since disclosure of sexual orientation/practices serves as a key determinant of vaccine recommendation, uptake is profoundly influenced by evidence suggesting that only 38% of MSM disclose this information to their HCPs.^31^ Similarly, disclosure of sexual orientation/practices pose unique challenges for transgender individuals, leading to missed opportunities to receive evidence-based preventative care (e.g., HPV vaccination).^32^ From this perspective, it is imperative that HCPs strive to create inclusive, trauma-informed and culturally-competent care environments, which are essential to fostering positive patient-provider relationships, trust in healthcare systems, and improving the uptake of recommended health interventions amongst underserved populations.^31,33^ Increasing efforts to mitigate the barriers influencing HCPs’ recommendation of inclusive HPV vaccination can bridge gaps in vaccine uptake and mitigate the persistence of HPV-related disparities in Canada.

### Accessing HPV Vaccination: Implications for Community-Based Programs

Despite the implementation of publicly funded school-based vaccination programs, HPV vaccine uptake amongst adolescents and young adults remains inconsistent, further reinforcing the need for robust community-based interventions.^6,17^ For example, inconsistent HPV vaccination coverage amongst adolescents has been attributed to concerns with parental consent, vaccine hesitancy and the exclusion of high-risk youth populations (e.g., unhoused youth).^34,35^ Additionally, newcomers to Canada represent another particularly underserved population, with approximately 70% reporting no prior awareness of HPV, and only 46% being aware of vaccination.^36^ Although HPV vaccination is widely available in medical clinics, pharmacies, community health centres and public health departments, access remains a significant barrier.^37^ A systematic review found that 38% of studies identified access as a primary obstacle to adult vaccination, highlighting an opportunity for stakeholders (e.g., researchers, community members, clinicians, policymakers) to develop tailored, community-centric solutions aimed at increasing HPV vaccine uptake amongst targeted, underserved populations (e.g., transgender peoples, MSM).^37^

### Enhancing HPV Vaccine Communication/Messaging

Given the complex landscape of HPV vaccination in Canada, multi-level communication strategies targeting HCPs, parents and eligible individuals are essential to addressing disparities in vaccine uptake.^38^ Evidence-based messaging- inclusive of public health vaccine campaigns- has been shown to increase vaccine acceptance, dispel misconceptions (e.g., safety concerns) and address hesitancy.^38^ However, operationalizing effective communication strategies remains a significant public health challenge.^38^ Community-based participatory research (CBPR) is an effective approach for integrating diverse perspectives into the design and implementation of health interventions, including vaccine promotion.^38^ By identifying beliefs, values and barriers to HPV vaccination amongst under-vaccinated populations, CBPR can ensure interventions are culturally and contextually appropriate.^38^ A one-size-fits-all approach to HPV vaccination fails to acknowledge the multi-level, intersectional factors influencing uptake, which continues to exacerbate vaccine-related disparities in Canada.^38^ By designing communication strategies that extend beyond the binary, gender-neutral approach, public health initiatives can effectively address disparities, making equitable vaccine uptake a tangible public health objective.

## Conclusion

In Canada, suboptimal HPV vaccine uptake remains a critical public health issue.^6^ While provincial and territorial school-based HPV vaccination programs effectively vaccinate a large proportion of adolescents, overall uptake remains below the NACI’s 90% target uptake goal.^6^ Current binary, gendered approaches to HPV vaccination pose significant challenges to equitably reducing HPV-related infections and associated diseases amongst underserved and under-vaccinated populations, including young adults, transgender peoples and MSM.

Addressing HPV vaccine-related disparities requires a coordinated, multi-level call to action involving collaboration between academic, clinical and community partners to normalize inclusive vaccination. This paper highlights various inequities in the distribution and uptake of HPV vaccination, which if left unaddressed, risks exacerbating vaccine-preventable morbidity and mortality. Achieving widespread HPV vaccine uptake depends upon various interrelated factors: strong HCP recommendation, accessible community-based vaccination services, and equitable representation of diverse populations in vaccine communication/messaging. Normalizing inclusive HPV vaccination is an urgent and necessary step that requires commitment at local, provincial/territorial and national levels to eliminate HPV-related diseases and strive towards global initiatives of health equity.

## Ethical Considerations

There are no human or animal participants recruited for this commentary and therefore, informed consent was not required.
